# *MINI BODY1*, encoding a MATE/DTX family transporter, affects plant architecture in mungbean (*Vigna radiata* L.)

**DOI:** 10.3389/fpls.2022.1064685

**Published:** 2022-11-17

**Authors:** Xin Li, Yahui Jia, Mingzhu Sun, Zikun Ji, Hui Zhang, Dan Qiu, Qiao Cai, Yan Xia, Xingxing Yuan, Xin Chen, Zhenguo Shen

**Affiliations:** ^1^ College of Life Sciences, Nanjing Agricultural University, Nanjing, China; ^2^ College of Agro-Grassland Science, Nanjing Agricultural University, Nanjing, China; ^3^ National experimental Teaching Center for Plant Production, Nanjing Agricultural University, Nanjing, China; ^4^ Institute of Industrial Crops, Jiangsu Academy of Agricultural Sciences, Nanjing, Jiangsu, China; ^5^ Jiangsu Collaborative Innovation Center for Solid Organic Waste Resource Utilization, Nanjing Agricultural University, Nanjing, China

**Keywords:** legume, mungbean, plant architecture, MIB1, MATE/DTX family, RNA-Seq

## Abstract

It has been shown that multidrug and toxic compound extrusion/detoxification (MATE/DTX) family transporters are involved in the regulation of plant development and stress response. Here, we characterized the *mini body1* (*mib1*) mutants in mungbean, which gave rise to increased branches, pentafoliate compound leaves, and shortened pods. Map-based cloning revealed that *MIB1* encoded a MATE/DTX family protein in mungbean. qRT-PCR analysis showed that *MIB1* was expressed in all tissues of mungbean, with the highest expression level in the young inflorescence. Complementation assays in *Escherichia coli* revealed that MIB1 potentially acted as a MATE/DTX transporter in mungbean. It was found that overexpression of the *MIB1* gene partially rescued the shortened pod phenotype of the *Arabidopsis dtx54* mutant. Transcriptomic analysis of the shoot buds and young pods revealed that the expression levels of several genes involved in the phytohormone pathway and developmental regulators were altered in the *mib1* mutants. Our results suggested that *MIB1* plays a key role in the control of plant architecture establishment in mungbean.

## Introduction

Plant architecture refers to the three-dimensional organization of plant organs, including the branching pattern and the shape and size of lateral organs, which affects plant growth and productivity (Reinhardt and Kuhlemeier, 2002; Wang and Li, 2008). During the last decades, multiple regulators in the control of plant architecture have been identified in model plants, such as rice (*Oryza sativa*) and *Arabidopsis thaliana*, which form complex regulatory networks including microRNA, key transcription factors, and phytohormones (Wang and Li, 2008; Guo et al., 2020).

The multidrug and toxic compound extrusion/detoxification (MATE/DTX) family was one of the important groups of multidrug transporters, which plays diverse roles in stress responses including detoxification, iron homeostasis, and drought stress (Diener et al., 2001; Li et al., 2002; Nawrath et al., 2002; Rogers and Guerinot, 2002; Magalhaes et al., 2007; Ishihara et al., 2008; Lu et al., 2019; Upadhyay et al., 2019; Duan et al., 2022; Nimmy et al., 2022). MATE/DTX family proteins also participate in plant development and growth (Thompson et al., 2010; Burko et al., 2011; Li et al., 2014; Suzuki et al., 2015; Jia et al., 2019; Upadhyay et al., 2020; Gani et al., 2022). For example, *Arabidopsis* ADP1/DTX51, a putative MATE/DTX family transporter, affects plant architecture. Elevated expression of *ADP1/DTX51* in *Arabidopsis* leads to an increase in plant growth rate and branch number by modulating the auxin level (Li et al., 2014). Another MATE/DTX transporter, BIG EMBRYO1 (BIGE1) in maize, regulates embryo development, initiation, and the size of lateral organs (Suzuki et al., 2015). The mutation of the maize *BIGE1* gene results in increased leaf number and larger embryo size. Similarly, the mutant of *DTX54/BIGE1A* (ortholog of *BIGE1* in *Arabidopsis*) exhibits increased leaf number and shortened pods with smaller seeds (Suzuki et al., 2015).

Legume is the third largest plant family, with more than 600 genus and 18,000 species (Graham and Vance, 2003). The plant architecture significantly affects the seed yield of grain legume such as pea (*Pisum sativa*), soybean (*Glycine max*), and mungbean (*Vigna radiata*). In pea, the TCP family gene *PsBRC1* integrates phytohormones including auxin, cytokinin (CK) and strigolactones (SL) to regulate shoot branching (Rameau et al., 2015; Kerr et al., 2021). It has been shown that the soybean gene *INCREASED LEAF PETIOLE ANGLE 1* (*GmILPA1*), encoding a subunit of the anaphase-promoting complex, controls the angle of leaf petiole (Gao et al., 2017). Notably, the *MicroRNA156* (*miR156*)-*SQUAMOSA PROMOTER BINDING PROTEIN-LIKE* (*SPL*) module has important roles in controlling plant architecture and agronomic traits in soybean (Bao et al., 2019; Sun et al., 2019). Overexpression of the *GmmiR156b* in soybean significantly alters plant architecture and improves seed yield (Sun et al., 2019). Consistently, knockout *GmmiR156b* targeted gene *GmSPL9* by gene editing alters plant architecture with improved performance and productivity in soybean (Bao et al., 2019). Recently, it has been shown that an MYB family transcription factor GmMYB14 in soybean regulates plant architecture through the brassinosteroid pathway. GmMYB14-overexpressing soybean plants display the compact plant architecture and improved seed yield (Chen et al., 2021). However, up to now, only a few key factors regulating plant architecture has been identified in legume and the underlying molecular mechanism is still poorly understood (Liu et al., 2020).

In this study, we characterized the *mini body1* (*mib1*) mutant in mungbean, which affected plant growth rate, branch number, and lateral organ size. It was found that *MIB1* encoded a member of MATE/DTX family proteins, potentially acting as a transporter in mungbean. Transcriptomic analysis revealed that expression levels of phytohormone pathway genes and developmental regulators were altered in the *mib1* mutants. Our results indicated that MIB1 plays a pivotal role in regulating plant architecture in mungbean.

## Materials and methods

### Plant materials

Three mutants, namely, *mib1-1* (A001), *mib1-2* (A006), and *mib1-3* (I007), were identified from M_2_ generation of the gamma ray mutagenized cultivar Sulu (Li et al., 2022). For phenotype analysis of wild-type (WT) plants, mutants were grown in the greenhouse at 28 ± 2°C, with a 16-h/8-h day/night photoperiod. The allelic tests for three mutants were carried out by crossing the *mib1-1* mutant with the *mib1-2* and *mib1-3* mutants, respectively. All plants of F_1_ generation showed the mutated phenotype.

### Scanning electron microscopy analysis

The terminal leaflets of the fifth compound leaves were fixed in FAA solution and then the samples were dehydrated in the ethanol/tert-butanol series. Field emission scanning electron microscopic (SU8010, Hitachi, Tokyo, Japan) analysis was conducted as previously described (Jiao et al., 2019).

### Map-based cloning of *MIB1* gene

The *mib1-3* mutants were crossed with cultivar AL127 to generate a population for genetic mapping. A total of 150 plants with mutant phenotype isolated from 642 plants in the F_2_ population were used to map the *MIB1* gene. The primers of the molecular markers used in present study are listed in **Supplementary Table 1**. The DNA were extracted *via* a plant Genomic DNA Kit DP305 (Tiangen, Beijing, China). The polymerase chain reaction (PCR) was carried out and the polymorphisms of the markers were analyzed as previously described (Jiao et al., 2016).

The PCR of the *MIB1* genomic region was conducted by the primers in **Supplementary Table 1**. The PCR products were cloned into the pMD18-T (TaKaRa, Dalian, China) and sequenced.

### RNA-sequencing analysis and quantitative reverse transcription PCR analysis

Shoot buds (2 weeks after germination) and the young pods (2 days after pollination) of WT and *mib1-3* mutants were collected with three biological replicates. RNA was extracted by the RNA Kit R6827-01 (Omega, Shanghai, China). We performed RNA-seq using the Illumina HiSeq X Ten platform (Illumina, San Diego, California, USA). The raw sequences were submitted to the NCBI SRA database with accession numbers SRR16944233–SRR16944244. Number of reads per kilobase of exon region in a gene per million mapped reads (RPKM) was used to value expression levels (Mortazavi et al., 2008), and VC1973A version 1.0 was used as the reference genome (Kang et al., 2014). Based on the methods described by Audic and Claverie (1997), DEGs were identified. Heat maps were generated by the pheatmap package (https://cran.r-project.org).

For qRT-PCR, the first strand cDNA was synthesized *via* Takara PrimeScript™ RT reagent Kit RR047A (TaKaRa, Dalian, China). qRT-PCR analysis was conducted using TB Green™ Premix Ex™ RR420A (TaKaRa) and the ABI StepOnePlus machine (Applied Biosystems, Foster City, CA, USA). Three biological replicates with three technical repeats were conducted.

### *Arabidopsis* transformation

The WT (Col-0) and *dtx54* mutant (WiscDsLoxHs046_04F) were used in the present study. The CDS of the *MIB1* gene was cloned into pCAMBIA1304 using primers in **Supplementary Table 1**. The construct was transformed into the *dtx54* mutants through floral dip transformation as previously described (Clough and Bent, 1998). T_3_ progeny lines of *35S::MIB1/dtx54* (L04 and L06) were used for phenotype analysis in this study.

### Complementation assays in *Escherichia coli*

WT strain K12 and *ΔacrB* mutant strain of *E. coli* were obtained from Professor Chuanzhen Jiang (South China Agricultural University). The CDS of the mungbean *MIB1* gene was cloned into the pET32a vector using primers in **Supplementary Table 1**, and the vectors were transformed into K12 and mutant strain. Transformants were selected on Luria-Bertani (LB) plate medium with 100 μg/ml ampicillin. The positive clones were then grown in liquid medium containing ampicillin and 1 mM isopropyl-β-D-thiogalactopyranoside (IPTG) to induce the expression of *MIB1*. The cells were diluted and spotted on medium plates with or without tetrabutyl ammonium (TBA) at 37°C for 24 h. Cell growth curves were determined by the absorbance at 600 nm of the cultures grown at 37°C for 24 h.

### Analysis of indole-3-acetic acid and abscisic acid contents

Plant hormone levels of indole-3-acetic acid (IAA) and abscisic acid (ABA) in young pods of the WT plant and mutants were determined by high-performance liquid chromatography–mass spectrum/mass spectrum (HPLC/MS/MS) by Agilent 1290 HPLC (Agilent, Santa Clara, CA, USA) and SCIEX-6500 Qtrap (AB Sciex, Foster, CA, USA), as described previously (Pan et al., 2010).

### Phylogenetic analysis

In this study, the MIB1 protein sequence was used to search against the mungbean database (Kang et al., 2014). The phylogenetic analysis was conducted by MEGA (version 7.0) using the neighbor-joining method with 1,000 replications (Kumar et al., 2016). The tree was displayed by the Interactive Tree of Life (iTOL; Letunic and Bork, 2016). Protein sequences from this study are listed in **Supplementary Table 2**.

## Results

### Isolation and characterization of the *mib1* mutants in mungbean

**Figure 1:**
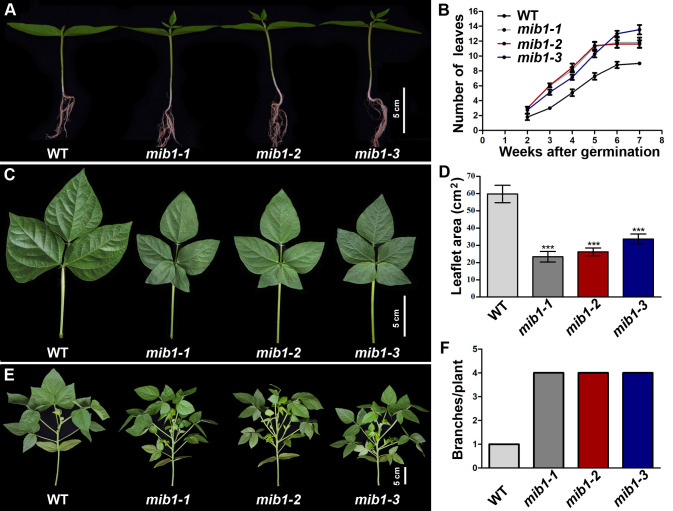
Growth rate characterization of WT and *mib1* mutants. **(A)** Two-week-old seedlings of the wild-type (WT) plant and *mib1* mutants. **(B)** The number of compound leaves of WT and *mib1* mutants (*n* = 10). **(C)** The fifth compound leaves of WT and *mib1* mutants. **(D)** The size of the terminal leaflets of the fifth compound leaves of WT and *mib1* mutants (*n* = 10). **(E)** Plant architecture of WT and *mib1* mutants at the 4 weeks after germination. **(F)** The number of branches of WT and *mib1* mutants at 4 weeks after germination (*n* = 10). The data were means ± SD. The Tukey’s multiple comparison test was used. *** *p* < 0.001.

To investigate key components regulating plant architecture in mungbean, we screened mutants with altered branch number and shape and size of lateral organs from the mutagenesis population (Li et al., 2022). Three allelic mutants affecting plant architecture were isolated in mungbean (**Figure 1**). We named these mutants *mini body1-1* (*mib1-1*), *mib1-2*, and *mib1-3*, respectively.

The leaf production rate in the *mib1* mutants was accelerated, compared with that of WT (**Figures 1A, B**). The juvenile leaves of the mutants were normal, but the adult leaves displayed pentafoliate form, compared to those of WT with trifoliate compound leaves (**Figure 1C**). In the *mib1* mutants, the size of the leaflets was severely reduced by 43.61%–60.93% (**Figure 1D**). The outgrowth of axillary buds in the *mib1* mutants was faster than those in WT (**Figure 1E**). The number of branches in the *mib1* mutants increased significantly (**Figure 1F**). At 4 weeks after germination, there was only one branch in each WT plant, while each *mib1* mutant had four branches (**Figure 1F**). At 8 weeks after germination, there was no difference in the number of primary branches between WT and mutants of *mib1-2* and *mib1-3* (the *mib1-1* mutant has about two more primary branches than WT; **Supplementary Figure 1**). However, the secondary branches in the three *mib1* alleles increased significantly (**Supplementary Figure 1**). Thus, the increased branch number in the mutants was caused by accelerated bud outgrowth and sustained branching capacity among early developed primary branches. Additionally, the *mib1* mutants had a compact plant architecture, compared with WT (**Figure 1E** and **Supplementary Figure 1A**).

**Figure 2:**
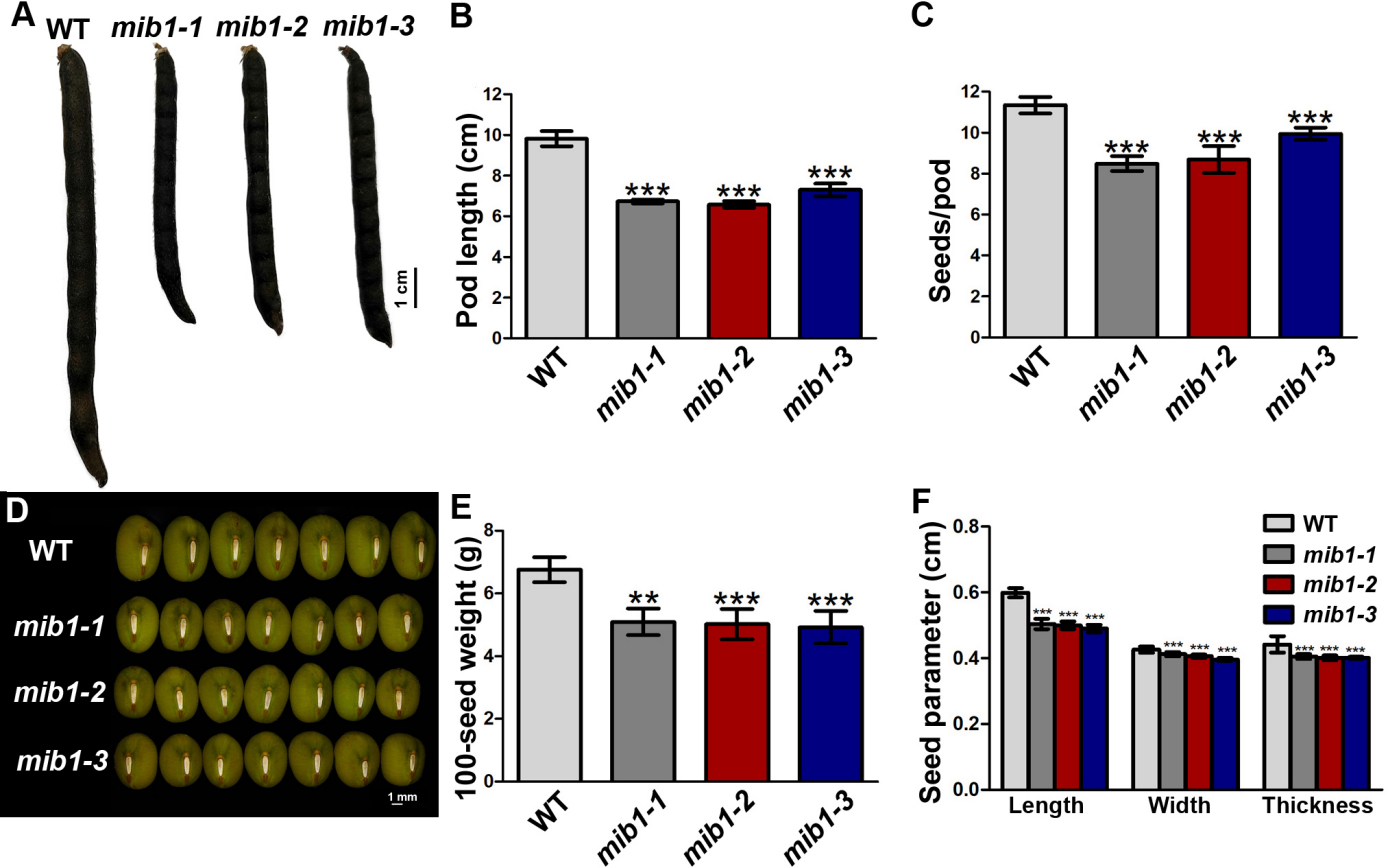
Characterization of pods and seeds of WT and *mib1* mutants. **(A)** Pods of WT and *mib1* mutants at matured stage. **(B)** Pod length of WT and *mib1* mutants (*n* = 150). **(C)** Seed number per pod of WT and *mib1* mutants (*n* = 150). **(D)** Seeds of WT and *mib1* mutants. **(E)** The 100-seed weights of WT and *mib1* mutants (*n* = 5). **(F)** Seed parameters of WT and *mib1* mutants (*n* = 200). The data were means ± SD. The Tukey’s multiple comparison test was used. ** *p* < 0.01, *** *p* < 0.001.

The flowers and young pods of the *mib1* mutants were smaller than those of WT (**Supplementary Figure 2**). The matured pods of the mutants were shorter, with decreased seed number and size (**Figures 2A–E**). The pod length of the *mib1* mutants (6.6± 0.03, 6.7± 0.05, and 7.3 ± 0.09 cm, respectively) was decreased, compared to that of WT (9.8 ± 0.11 cm). The seed number per pod of three *mib1* mutants (8.5 ± 0.11, 8.7 ± 0.20, and 9.9 ± 0.09, respectively) was much lower than that of WT (11.3 ± 0.65). Compared with the WT, mature seeds of *mib1* mutants were rounder and showed significantly decreased length, width, and thickness (**Figures 2D, F**). Therefore, the 100-seed weight was decreased by 26.82%, 18.63%, and 27.42% in *mib1-1*, *mib1-2*, and *mib1-3* mutants, compared with that of WT, respectively (**Figure 2E**).

The plant organ size is regulated by the coordination of two connected processes, cell division and expansion (Gonzalez et al., 2012). Microscopic examination of leaflet epidermal cells showed that the cell size decreased significantly in the *mib1-3* mutants in comparison with that of WT (**Supplementary Figure 3A**). The area of epidermal cells in the *mib1-3* mutants was only about half that in the WT plants (**Supplementary Figure 3B**), suggesting that *MIB1* augments organ size mainly by increasing the cell size.

### Molecular characterization of the *MIB1* gene in mungbean

Genetic analysis of the *mib1* mutants was conducted by backcrossing *mib1-3* mutants with the WT plants. All F_1_ plants were similar to WT. In the F_2_ population, the WT plants and mutant plants segregated with a 3:1 ratio (87 WT plants and 25 mutants, *χ* ^2^ = 0.42 < *χ* ^2^_0.05_ = 3.84), indicating that *mib1* was a single recessive locus.

**Figure 3:**
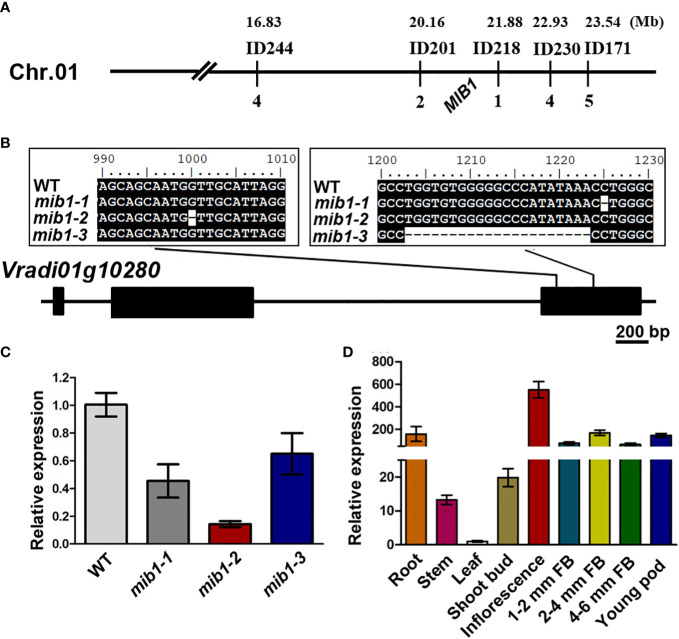
Map-based cloning of *MIB1*. **(A)** Genetic map of *MIB1* in mungbean. **(B)** Mutations in the open reading frame of *Vradi01g10280*. Numbers up the sequence indicate the position on the open reading frame. **(C)** Analysis of *MIB1* expression in shoot buds of WT and *mib1* mutant by qRT-PCR. **(D)** Relative expression level of *MIB1* in different tissues of WT.

We conducted map-based cloning to identify the *MIB1* gene (Jiao et al., 2016). The *MIB1* gene was preliminarily mapped on chromosome 1 of the VC1973A genome (Kang et al., 2014), linked with the markers ID244 and ID171 (**Figure 3A**). By developing new markers, the *mib1* mapping region was narrowed down to a 1.71-Mb region flanked by the markers ID218 and ID201 (**Figure 3A**). Based on the functional annotation (Kang et al., 2014) and the mutant phenotype, *Vradi01g10280* (*LOC106766026*) in the mapping region was identified as the candidate (**Figure 3B**). Sequencing of the PCR products of *Vradi01g10280* from WT and *mib1* mutants displayed mutations (**Figure 3B**), showing that three alleles, *mib1-1*, *mib1-2*, and *mib1-3*, carried different deletions (1-bp deletion, 1-bp deletion, and 21-bp deletion, respectively). qRT-PCR analysis of shoot buds (2 weeks after germination) revealed that there were decreased expression of the *Vradi01g10280* gene in the *mib1* mutants (**Figure 3C**).

**Figure 4:**
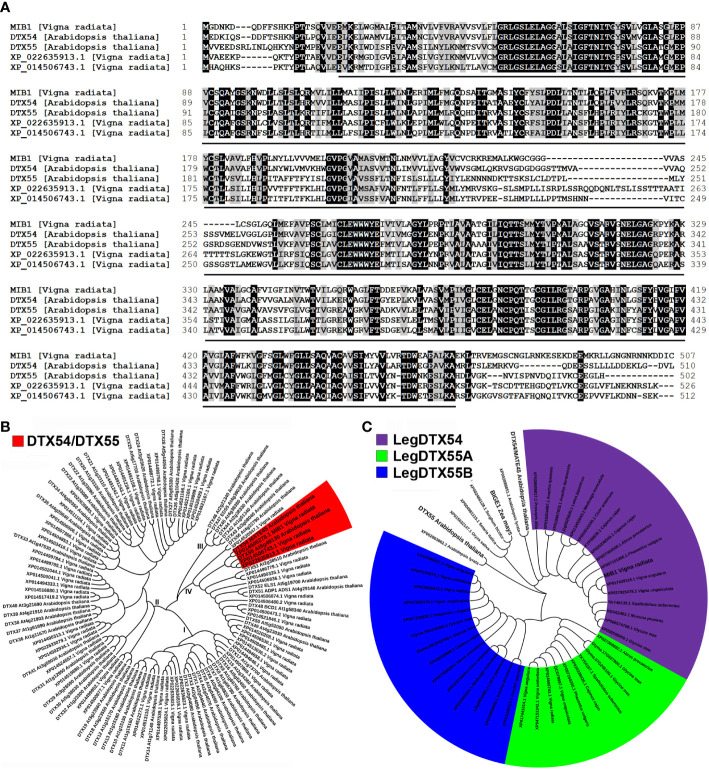
Analysis of the MIB1 protein isolated from mungbean. **(A)** Alignment of amino acid sequences of MIB1, DTX54, DTX55, XP_022635913.1, and XP_014506743.1. The conserved MatE domain was indicated by the black line. **(B)** Phylogenetic tree of MATE family proteins in mungbean and *Arabidopsis*. I, II, III, and IV represent four different groups. **(C)** Phylogenetic analysis of LegDTX54 clade and LegDTX55 clade proteins in legume.

Segregation analysis showed that 150 mutated plants out of a total of 642 individuals from the F_2_ mapping population were homozygous for the 21-bp deletion in *Vradi02g10020*, indicating that the deletion co-segregates with the mutant phenotype. Therefore, *MIB1* (*Vradi01g10280*) encoded a member of MATE/DTX proteins (**Figure 4**), which was closely related to DTX54/BIGE1A in *Arabidopsis* and BIGE in maize (Suzuki et al., 2015), affecting plant architecture in mungbean.

The relative expression of the *MIB1* gene in different tissues of mungbean was analyzed by qRT-PCR. It was found that the *MIB1* gene was expressed in all tissues, with the highest expression level in the young inflorescence (**Figure 3D**).

### MIB1 was a member of the MATE/DTX family proteins in mungbean

Multiple amino acid sequence alignments of the MIB1 protein (XP_014506278.1) with its homologs indicated that it shared a conserved MatE domain (**Figure 4A**). The MIB1 protein was predicted to have 12 transmembrane domains with N-termini towards the inside of the cell (**Supplementary Figure 4**).

We conducted a BLASTP search for sequences with homology to MIB1 to characterize the MATE/DTX family in the mungbean database (Kang et al., 2014) and found 56 MATE/DTX proteins in the mungbean genome (**Supplementary Table 2**). These mungbean MATE/DTX proteins were classified into four groups by phylogenetic analysis with *Arabidopsis* MATE/DTX proteins (**Figure 4B**; Wang et al., 2016). It was found that MIB1 had two other closely related homologs in mungbean, XP_022635913.1 (Vradi05g00900) and XP_014506743.1 (Vradi07g25110, **Figure 4B**).

In order to investigate the origin of MIB1 in legume plants, we identified MIB1 closed homologs from a number of public databases (**Supplementary Table 2**). The phylogenetic tree of aligned legume DTX54 and DTX55 orthologs was constructed (**Figure 4B**). It was found that one copy encoding the ortholog to DTX54 in legume formed the LegDTX54 clade, which was distinct from the LegDTX55 clade (**Figure 4C**). In contrast, within the LegDTX55 clade, there were different copies in legume, such as two copies in adzuki bean (*V. angularis*) and mungbean, and one copy in *Medicago truncatula* and *Lotus japonicus* (**Figure 4C**). The best phylogeny places the legume DTX55A (LegDTX55A) subclade and the legume DTX55B (LegDTX55B) subclade sister together, forming the LegDTX55 clade in legume (**Figure 4C**).

### Heterologous expression of mungbean *MIB1* gene increased TBA tolerance in the mutant *Escherichia coli*

**Figure 5:**
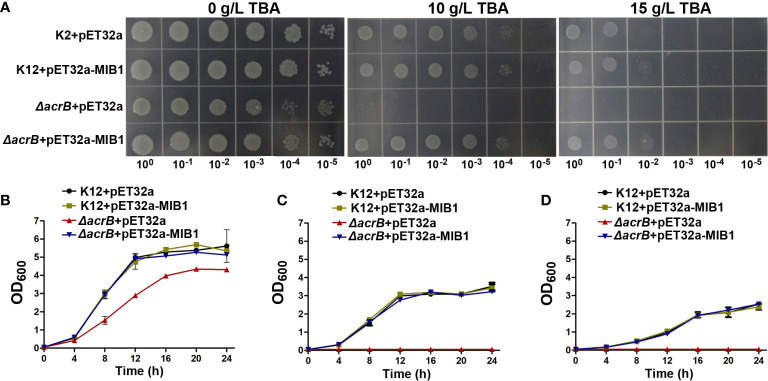
Analysis of MIB1 transport function in *Escherichia coli.* **(A)** The effect of *MIB1* expression on the growth of (*E*) *coli* cells under TBA treatment on the LB plate. (*E*) *coli* cells were spotted on the LB plate with 0, 10, and 15 g/L TBA for 24 h. 10^0^, 10^−1^, 10^−2^, 10^−3^, 10^−4^, and 10^−5^ represented dilution series. **(B–D)** The effect of *MIB1* expression on the growth curve of (*E*) *coli* cells under TBA treatment*. (E) coli* cells were inoculated in liquid LB medium with 0 **(B)**, 10 **(C)**, and 15 **(D)** g/L TBA for 24 h. The data were means ± SD (*n* = 3).

To investigate the functional character of the MIB1 protein, the expression vector containing the *MIB1* gene or empty vector was transformed into the WT strain (K12) and mutant strain (*ΔacrB)* in *E. coli*. The *ΔacrB* mutant strain lacks the functional AcrAB complex, the multidrug efflux carrier (Seo et al., 2012), and cannot grow under toxic conditions. The transformed cells were grown on the medium with and without different concentrations of TBA. The *ΔacrB* mutant cells with empty expressing vector (pET32a) did not grow on an LB plate with 10 and 15 mg/ml TBA (**Figure 5A**), while the *MIB1*-expressing *ΔacrB* cells continued their growth on the LB medium with 10 and 15 mg/ml TBA (**Figure 5A**), suggesting that MIB1 in mungbean potentially acts as a MATE/DTX family transporter.

In order to further verify the results of the plate experiment, we determined the growth curve of the strains under 0, 10, and 15 mg/ml TBA treatment in liquid LB medium (**Figures 5B–D**). Compared to those of expressing *MIB1* cells and the WT strain, TBA treatment significantly inhibited the growth of the mutant strain (**Figures 5B–D**). Under 10 and 15 mg/ml TBA treatments for 24 h, the growth curve of the mutant strain expressing *MIB1* was similar to those of the WT strain with and without expressing *MIB1* (**Figures 5C, D**). The above results showed that heterologous expression of mungbean *MIB1* increased TBA tolerance of the *ΔAcrB* mutant strain.

### Heterologous expression of mungbean *MIB1* gene partially rescued the pod phenotype of *dtx54* mutant in *Arabidopsis*

**Figure 6:**
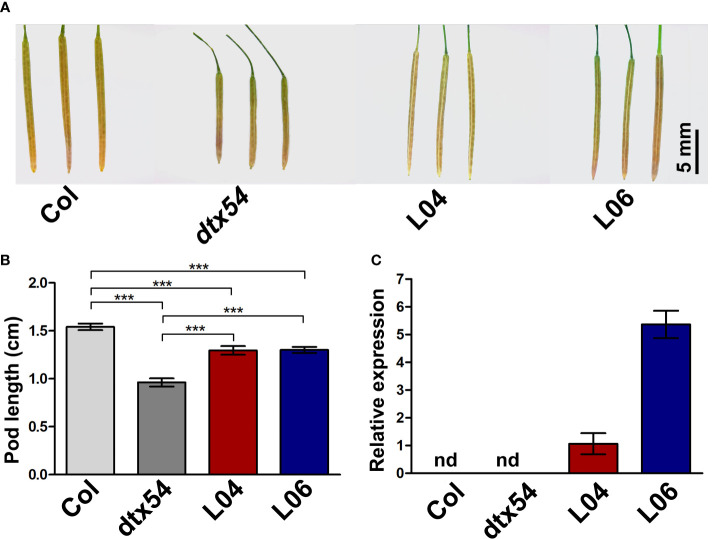
*MIB1* partially rescued the shortened pod phenotype of *Arabidopsis dtx54* mutant. **(A)** The pod phenotype of the wild-type plant (Col), *dtx54* mutant, and *35S::MIB1* transgenic lines of *dtx54* (L04 and L06). **(B)** The pod length of Col, *dtx54* mutants, and two transgenic lines (*n* = 100). **(C)** qRT-PCR analysis of *MIB1* expression from Col, *dtx54* mutant, and two transgenic lines. nd, not detected. The data were means ± SD. One-way ANOVA was used. ***p < 0.001.

It has been reported that loss of function of *DTX54/BIGE1A*, *MIB1* ortholog in *Arabidopsis*, leads to the shortened pods (Suzuki et al., 2015). To test whether *MIB1* performs a similar function to *DTX54/BIGE1A* in the control of pod development, the coding sequence of *MIB1* driven by the cauliflower mosaic virus 35S (CaMV35S) promoter was transformed into the *Arabidopsis dtx54* mutant (*35S::MIB1/dtx54*, two representative lines L04 and L06). It was found that the shortened pods were partially rescued in *35S::MIB1/dtx54* transgenic lines (**Figures 6A, B**). The expression of *MIB1* was detected in *35S::MIB1/dtx54* transgenic lines (**Figure 6C**). The above results indicated that the mungbean *MIB1* gene plays a conserved role in the pod development.

### RNA-seq analysis of the wild-type plants and *mib1* mutants

**Figure 7:**
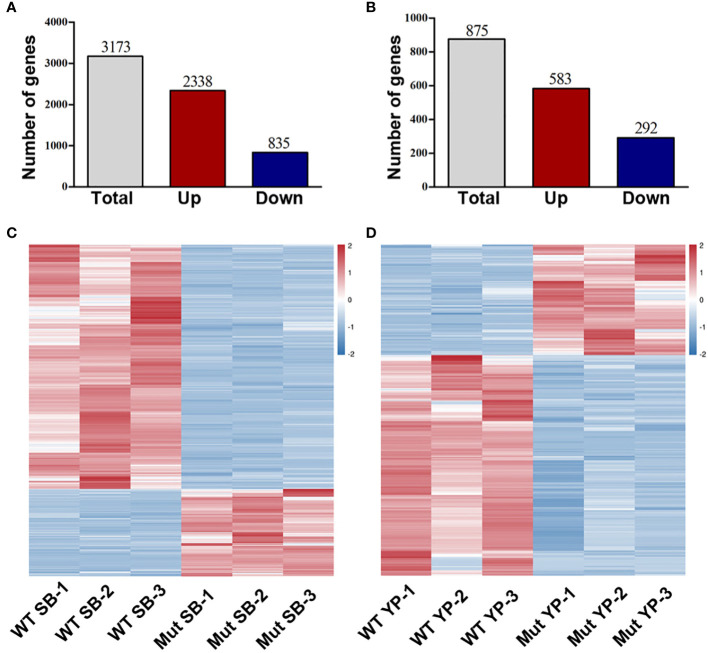
RNA-seq analysis of DEGs between WT and *mib1* mutants. **(A)** Number of DEGs of the shoot buds between WT and *mib1* mutants. **(B)** Number of DEGs of the young pods between WT and *mib1* mutants. **(C)** Heat map of the DEGs of the shoot buds between WT and *mib1* mutants. **(D)** Heat map of the DEGs of the young pods between WT and *mib1* mutants.

**Figure 8:**
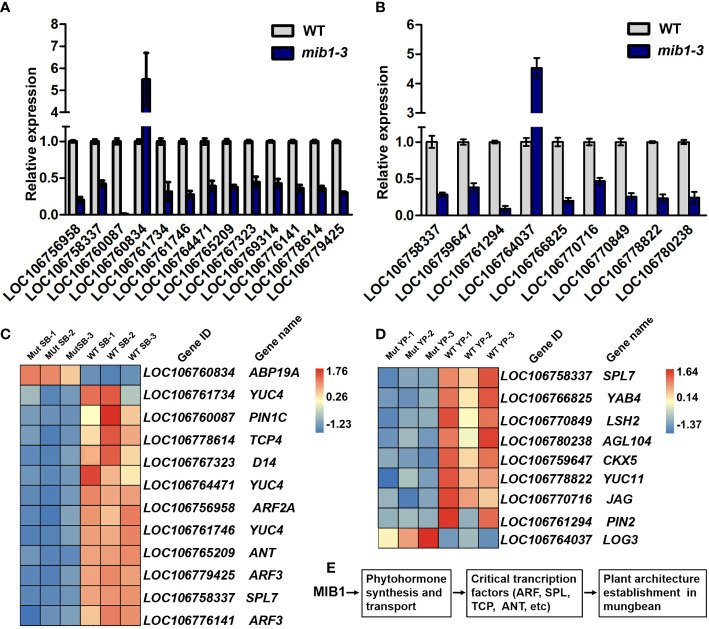
Critical DEGs involved in the control of plant architecture in mungbean. **(A)** qRT-PCR analysis of 13 DEGs in the shoot buds between WT and *mib1* mutants. **(B)** qRT-PCR analysis of eight DEGs in young pods between WT and *mib1* mutants. **(C)** Heat map showing critical DEGs involved in the control of the rate of leaf production, branch number, and organ size. **(D)** Heat map showing critical DEGs in involved young pod development. **(E)** A hypothetical model of MIB1 affecting plant architecture in mungbean.

To investigate the potential genes whose expression was altered in the mutants, RNA-sequencing (RNA-seq) analysis was conducted to compare the gene expression levels in shoot buds and young pods between WT and *mib1-3* mutants. A total of 3,173 and 875 differentially expressed genes (DEGs) were identified at the two developmental stages, respectively (**Figure 7**, **Supplementary Tables 3** and **4**). The qRT-PCR analysis confirmed the results of the RNA-Seq (**Figures 8A, B**).

Consistent with the mutant phenotype of plant architecture, the expression levels of key components of the plant hormone pathway and transcription factors related to plant development and growth were changed in the *mib1* mutants (**Figures 8C, D**). Among DEGs of the shoot buds between WT and *mib1* mutants, three auxin biosynthesis genes (*LOC106761734*, *LOC106761746*, and *LOC106764471*, *YUCCA 4*, *YUC4*) and a gene encoding auxin transporter (*LOC106760087*, *PIN-FORMED 1C*, *PIN1C*) were downregulated (**Figure 8C**). Several transcription factor encoding genes involved in plant development, such as *LOC06756958* (*Auxin Response Factor 2*, *ARF2*), *LOC106778614* (TCP family gene, *TCP4*), *LOC106769314* and *LOC106758337* (SPL family genes, *SPL7* and *SPL8*), and *LOC06765209* (AP2/ERF family gene *AINTEGUMENTA*, *ANT*), were downregulated (**Figure 8C**). Additionally, *LOC106767323* (*DWARF 14*, *D14*), encoding a key component of the SL signaling pathway (Zhou et al., 2013), was downregulated in the shoot buds of the *mib1* mutants (**Figure 8C**).

It has been shown that auxin and cytokinin pathways play a key role in the control of pod development and seed number per pod (Liu et al., 2021; Qadir et al., 2021; Yu et al., 2022). We found that the auxin biosynthesis gene (*LOC106778822*, *YUC11*) and the auxin transporter encoding gene (*LOC106761294*, *PIN2*) were downregulated in the young pods of the *mib1* mutants (**Figure 8D**). Consistently, there was a significant reduction in IAA level in young pods of the *mib1* mutants, compared to that of WT (**Supplementary Figure 5**). In addition, the expression levels of *LOC106759647* (*Cytokinin dehydrogenase 3*, *CKX3*) and *LOC106764037* (*LONELY GUY 3*, *LOG3*), related to the cytokinin pathway, were also significantly changed in the young pods of the *mib1* mutants (**Figure 8D**).

## Discussion

### *MIB1* encoded a MATE/DTX family transporter, affecting plant architecture in mungbean

The plant architecture significantly affects the seed yield of grain legume. However, the underlying molecular mechanism is still poorly understood (Liu et al., 2020). In this study, The mutations of the *MIB1* gene in mungbean resulted in bushy and compact plant architecture (**Figure 1**) and shortened pods with smaller and rounder seeds (**Figure 2**). Map-based cloning showed that the *MIB1* gene encoded a MATE/DTX family protein in mungbean, which was an ortholog of DTX54/BIGE1A in *Arabidopsis* and BIGE in maize (**Figure 4**). It has been reported that loss of function of the *DTX54/BIGA1A* gene, *MIB1* ortholog in *Arabidopsis*, gives rise to increased branch numbers and shortened pods (Suzuki et al., 2015). We found that heterologous expression of the *MIB1* gene partially rescued the phenotype of *dtx54/bige1a* mutant in *Arabidopsis*, suggesting that MIB1 plays a conserved role in the control of pod development.

MIB1 belonged to group IV of the MATE/DTX family (**Figure 4A**). Complementation assays in *E. coli* showed that MIB1 potentially acted as a MATE/DTX transporter in mungbean. Meanwhile, there was a significant reduction in IAA levels in young pods of the *mib1* mutants (**Supplementary Figure 5**). Consistently, transcriptome analysis revealed that expression levels of the genes related to auxin synthesis and transport were decreased (**Figure 8**). Thus, our results suggested that auxin plays a key role in regulating plant architecture in mungbean. The alteration of plant architecture in the *mib1* mutants was probably due to the modulated levels of auxin and other plant hormones and then the altered expression of the downstream genes related to plant growth and development (**Figure 8E**).

It has been reported that the group IV MATE/DTX transporters are able to modulate plant hormone levels such as auxin and ABA in *Arabidopsis* and rice (Li et al., 2014; Zhang et al., 2014; Qin et al., 2021). Thus, how plant hormone level is modulated by the MIB1 protein should be investigated in more detail in the future.

### Phylogenetic analysis of DTX54 and DTX55 orthologs in legume

It has been shown that the *DTX54/BIGE1A* and *DTX55/BIGE1B* in *Arabidopsis*, two paralogs, have partial functional redundancy and diversity (Suzuki et al., 2015). The mutant of the *Arabidopsis DTX54/BIGE1A* gene shows increased number of leaves (Suzuki et al., 2015). By contrast, the *dtx55* mutant exhibits a slight increase in leaf number, suggesting that *Arabidopsis DTX5*4 has a greater role in the control of leaf initiation, while the leaf number of the double mutants of *DTX54/BIGE1A* and *DTX55/BIGE1B* is enhanced compared to the single mutants, indicating that there is an additive interaction between the two genes (Suzuki et al., 2015).

It was found that there were 56 MATE/DTX family proteins in mungbean genome (**Figure 4**), among which two other MATE/DTX proteins are closely related to MIB1 and might redundantly affect plant development and growth in mungbean. Moreover, based on the public sequences, we identified DTX54 and DTX55 orthologs in legume. We found that there was a single copy encoding the DTX54 orthologs in legume (**Figure 4**). In contrast, the legDTX55 clade in legume could be further divided into two subclades, LegDTX55A and LegDTX55B (**Figure 4B**).

At present, mutant libraries for several legume species such as *M. truncatula*, *L. japonicus*, and *G. max* are available (Tadege et al., 2008; Małolepszy et al., 2016; Li et al., 2017), and it would be worth identifying the mutant lines of the LegDTX54 and LegDTX55 clade genes in these species to dissect their function in plant architecture establishment. Moreover, it is also interesting to study the interactions between the LegDTX54 clade and LegDTX55 clade genes during plant development and growth in legume.

## Data availability statement

The datasets presented in this study can be found in online repositories. The names of the repository/repositories and accession number(s) can be found in the article/**Supplementary Material**.

## Author contributions

XL wrote the manuscript. XL, YJ, MS, ZJ, HZ, DQ, QC, YX, and XY performed the experiments. XC and ZS supervised the research. XL and ZS analyzed the data and prepared the manuscript. All authors contributed to the article and approved the submitted version.

## Funding

This research was funded by the Science Foundation of Jiangsu Province, China (BE2021718), Jiangsu Seed Industry Revitalization Project (JBGS[2021]004), the Jiangsu Agricultural Science and Technology Innovation Fund of China (CX(20)3030), and the Students' innovation and entrepreneurship training program of National experimental Teaching Center for Plant Production (ZKF202212).

## Acknowledgments

We would like to thank Professor Chuanzhen Jiang (South China Agricultural University) for providing the K12 and *ΔacrB* mutant strains.

## Conflict of interest

The authors declare that the research was conducted in the absence of any commercial or financial relationships that could be construed as a potential conflict of interest.

## Publisher’s note

All claims expressed in this article are solely those of the authors and do not necessarily represent those of their affiliated organizations, or those of the publisher, the editors and the reviewers. Any product that may be evaluated in this article, or claim that may be made by its manufacturer, is not guaranteed or endorsed by the publisher.

## Supplementary material

Supplementary Figure 1 The number of primary and secondary branches of WT and *mib1* mutants at the matured stage. **(A)** Plant architecture of WT and *mib1* mutants at the 8 weeks after germination; **(B)** The number of primary and secondary branches of WT and *mib1* mutants. The data were means ± SD (n=10). The Tukey’s multiple comparison test was used. ***p* < 0.01, ****p* < 0.001.

Supplementary Figure 2 Characterization of the flowers **(A)** and young pods **(B)** of WT and *mib1* mutants.

Supplementary Figure 3 SEM analysis of the terminal leaflets of WT and *mib1* mutants. **(A)** The epidermal cells of the terminal leaflets of the fifth compound leaves in WT and *mib1-3* mutants; **(B)** The size of epidermal cells from the terminal leaflets of WT and *mib1-3* mutants. The data were means ± SD (n=100). The Student’s test was used. ****p* < 0.001.

Supplementary Figure 4 The 12 transmembrane domains of the MIB1 protein was predicted using the DeepTMHMM. **(A)** Most likely topology of MIB1; **(B)** Posterior probabilities of MIB1.

Supplementary Figure 5 The hormone levels of IAA and ABA in young pods of WT and *mib1* mutants. The data were means ± SD (n=5). The Student’s test was used. ***p* < 0.01.

Supplementary Table 1 Primer list used in this study.

Supplementary Table 2 MATE family proteins used for phylogenetic analysis in this study.

Supplementary Table 3 DEGs in shoot buds between WT and *mib1* mutants.

Supplementary Table 4 DEGs in young pods between WT and *mib1* mutants.
