# Supplementary figures and images for: MINI BODY1, encoding a MATE/DTX family transporter, affects plant architecture in mungbean (Vigna radiata L.)
Source: Front Plant Sci. 2022 Nov 17;13:1064685. doi: 10.3389/fpls.2022.1064685 (PMC9714821; doi:10.3389/fpls.2022.1064685)

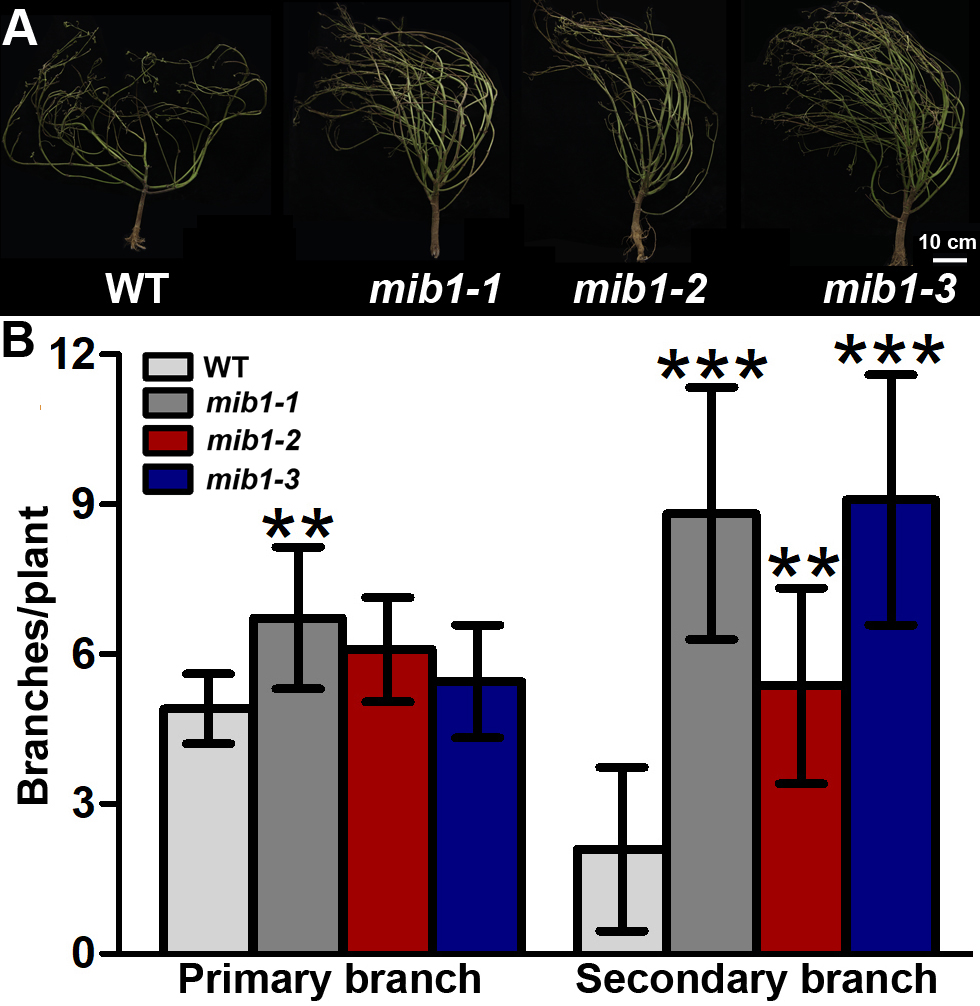

Supplement: Supplementary Figure 1 — The number of primary and secondary branches of WT and mib1 mutants at the matured stage. (A) Plant architecture of WT and mib1 mutants at the 8 weeks after germination; (B) The number of primary and secondary branches of WT and mib1 mutants. The data were means ± SD (n=10). The Tukey’s multiple comparison test was used. **p < 0.01, ***p < 0.001. [file Image_1.jpeg]

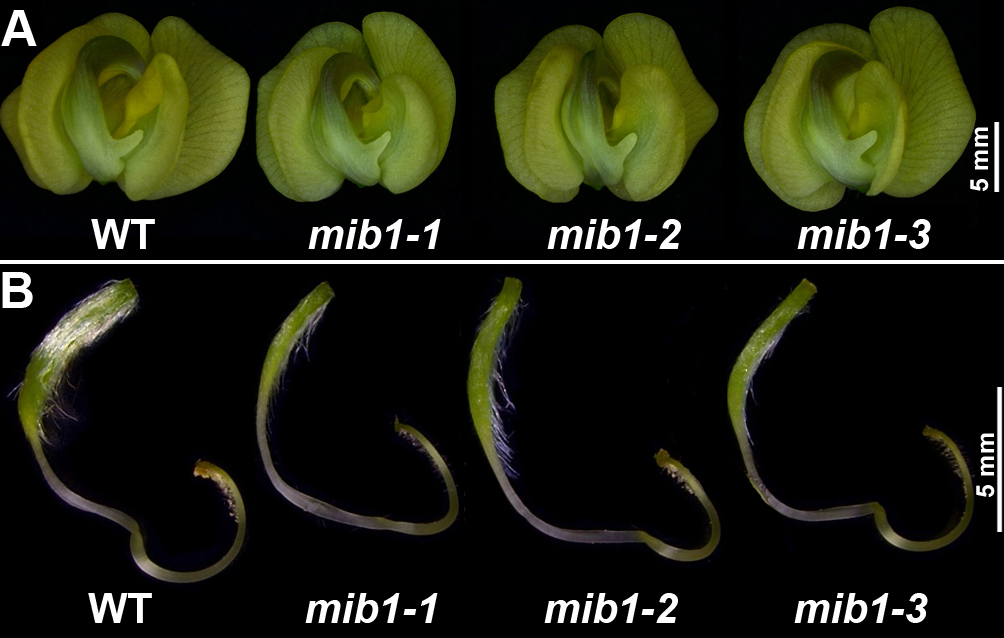

Supplement: Supplementary Figure 2 — Characterization of the flowers (A) and young pods (B) of WT and mib1 mutants. [file Image_2.jpeg]

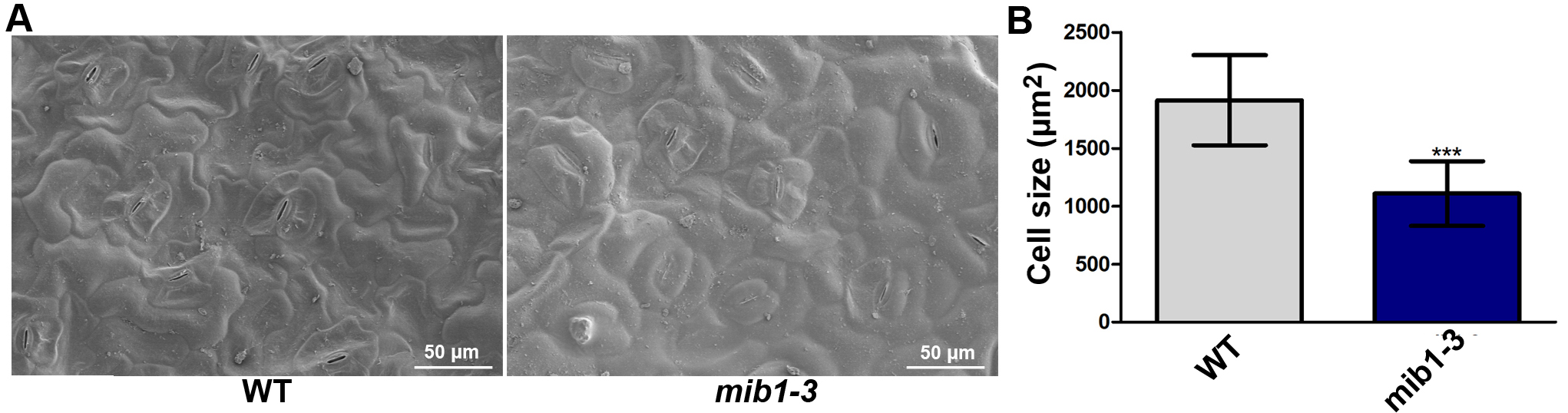

Supplement: Supplementary Figure 3 — SEM analysis of the terminal leaflets of WT and mib1 mutants. (A) The epidermal cells of the terminal leaflets of the fifth compound leaves in WT and mib1-3 mutants; (B) The size of epidermal cells from the terminal leaflets of WT and mib1-3 mutants. The data were means ± SD (n=100). The Student’s test was used. ***p < 0.001. [file Image_3.jpeg]

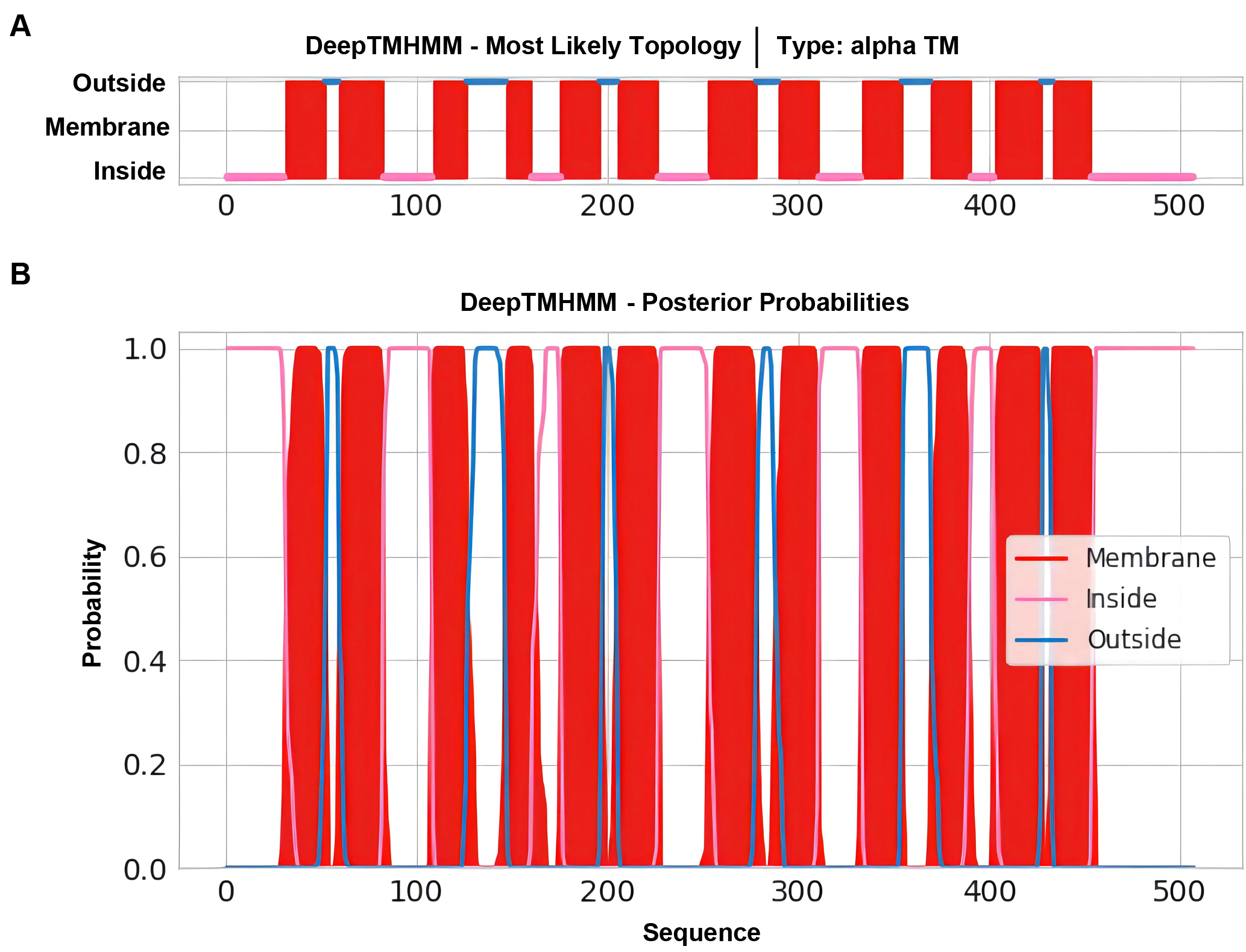

Supplement: Supplementary Figure 4 — The 12 transmembrane domains of the MIB1 protein was predicted using the DeepTMHMM. (A) Most likely topology of MIB1; (B) Posterior probabilities of MIB1. [file Image_4.jpeg]

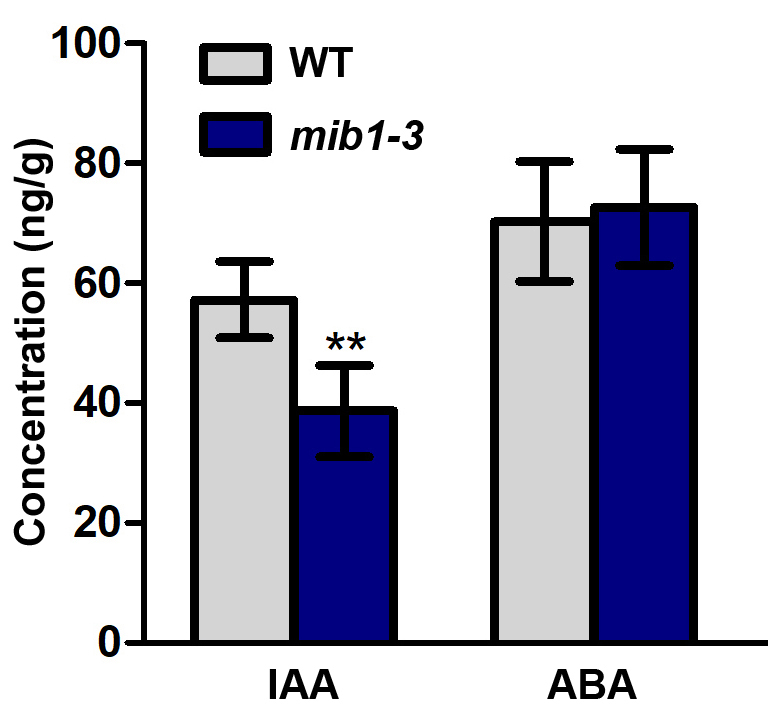

Supplement: Supplementary Figure 5 — The hormone levels of IAA and ABA in young pods of WT and mib1 mutants. The data were means ± SD (n=5). The Student’s test was used. **p < 0.01. [file Image_5.jpeg]
